# Confounding factors in article stating that Ubiquitin C terminal hydrolase predicts poor neurological outcome after cardiac arrest

**DOI:** 10.1186/s13054-023-04462-1

**Published:** 2023-05-15

**Authors:** Patrick M. Honoré, Emily Perriens, Ibrahim Bousbiat, Nahida Harim, Elena Germain, Patrick El Nawar, Sydney Blackman

**Affiliations:** 1 ICU Department, CHU UCL Godinne Namur, UCLouvain Medical School, Avenue G Thérasse 1, 5530 Yvoir, Belgium; 2 ICU Brugmann University Hospital, ULB University, Brussels, Belgium; 3 ULB University, Brussels, Belgium

**Keywords:** Ubiquitin C, Correlation with poor neurological outcome, Potential confounders

Song et al. conclude that novel serum biomarkers predict poor neurological outcome after cardiac arrest (CA) with high accuracy [1]. Cutoffs from two large existing studies (TTM and COMACARE substudy) were externally validated in their study [1]. According to Song et al., the predictive power of novel biomarkers was the highest 72 h after CA [2].

In their analysis, the predictive performance of Ubiquitin C terminal hydrolase (UCHL) was seen in patients with poor outcome [1]; higher values of UCHL were observed in these patients [1]. Nearly half of critically ill patients—especially those with shock—had or developed acute kidney injury (AKI) and 20–25% needed renal replacement therapy (RRT) within the first week of hospitalization [2]. In Song’s study, the out-of-hospital cardiac arrest (OHCA) group was very sick on admission with lactate between 6 and 9 mmol/L [1]. Therefore, we could make the assumption that 20–25% of these patients would require RRT or continuous RRT (CRRT). As the study did not provide numbers regarding RRT, this assumption may also overestimate any negative impact on the estimated effect. UCHL’s molecular weight of 25 kDa makes it theoretically very easily removable by RRT and CRRT [3]. Although theoretically possible, there is little to no published data on this issue. CRRT is performed using membranes with a cut off of 35–40 kDa; it is, therefore, logical to assume that a potential portion of UCHL is eliminated by the CRRT [4]. New highly adsorptive membranes (HAMs) are able to adsorb molecules with molecular weights greater than 35 kDA, further increasing the removal of UCHL [5]. Not taking into account the effect of RRT and CRRT on UCHL can mislead evaluations and conclusions by artificially reducing the level of UHCL and underestimating its effects in each group (good versus poor neurological outcome) [1]. Nevertheless, only a study looking into UCHL clearance could precisely quantify the loss of it by RRT and the potential impact on the results of the study. If the findings of this new study show that UCHL is significantly removed by RRT, it is necessary to exclude patients with AKI that may need RRT or CRRT to avoid potentially underestimating the levels of UCHL in each group (good versus poor neurological outcome) undergoing RRT or CRRT.

## Data Availability

Not applicable.

## Abbreviations

CA: Cardiac arrest
UCHL: Ubiquitin C terminal hydrolase
AKI: Acute kidney injury
RRT: Renal replacement therapy
CRRT: Continuous RRT
